# Intestinal Sarcoidosis: A Diagnostic Conundrum

**DOI:** 10.7759/cureus.88290

**Published:** 2025-07-19

**Authors:** Alveena Imran, Rida Suleman, Rahul Bhat, Humayun Nasir

**Affiliations:** 1 Respiratory Medicine, George Eliot Hospital NHS Trust, Nuneaton, GBR; 2 Medicine, George Eliot Hospital NHS Trust, Nuneaton, GBR

**Keywords:** colon sarcoidosis, extrapulmonary sarcoidosis, gi sarcoidosis, intestinal sarcoidosis, large intestine sarcoidosis

## Abstract

Sarcoidosis is a systemic granulomatous disorder of unknown cause, most commonly affecting the lungs and intrathoracic lymph nodes. We present the case of a 51-year-old woman in whom intestinal sarcoidosis was incidentally discovered alongside pulmonary involvement. The diagnosis was made during evaluation of trauma-related findings and confirmed through PET-CT, colonoscopy, and biopsy. GI sarcoidosis can closely mimic other granulomatous diseases, such as Crohn’s disease or certain infections, making diagnosis particularly challenging. In this case, the presence of mediastinal lymphadenopathy with granulomas and PET-positive uptake in the ascending colon and a lymph node supported the diagnosis. Infectious and inflammatory bowel disease causes were excluded. The patient remained asymptomatic, so treatment was not initiated, consistent with current guidelines that recommend corticosteroids or surgical intervention only for symptomatic or severe disease. Intestinal sarcoidosis, particularly colonic involvement, remains exceedingly rare. However, clinicians should consider it in the differential diagnosis of colonic lesions, especially when imaging reveals evidence of extraintestinal sarcoidosis. Diagnosis relies on histopathological confirmation and the exclusion of other potential causes. Many patients remain asymptomatic and do not require treatment, although corticosteroids, immunomodulators, or surgery may be necessary in symptomatic or complicated cases. Further research is warranted to explore potential associations, such as with gastroesophageal reflux disease, and to determine optimal management strategies.

## Introduction

Sarcoidosis is an inflammatory disorder of unknown etiology that primarily affects the lungs and intrathoracic lymph nodes. It is thought to result from an exaggerated immune response to an unidentified antigen; however, the exact cause remains unknown. The hallmark of the disease is the presence of noncaseating granulomas in affected tissues. While pulmonary involvement is most common, sarcoidosis can also affect the brain, skin, eyes, kidneys, heart, and GI tract [1].

According to the literature, GI involvement most frequently occurs in the upper tract - including the esophagus, stomach, and duodenum - and rarely involves the colon. Reported colonic cases typically show polyps, nodules, ulcers, strictures, or obstruction on endoscopic examination [2].

We report the case of a 51-year-old woman diagnosed with intestinal sarcoidosis as an extrapulmonary manifestation, alongside pulmonary sarcoidosis. This case underscores the importance of including sarcoidosis in the differential diagnosis when radiographic findings suggest metastatic disease.

## Case presentation

A 51-year-old female doctor of South Asian origin presented to the ED of a district general hospital following a road traffic accident, with mild neck and chest pain and abdominal tenderness. There was no history of fever, skin rash, visual disturbances, joint pain, cough, breathlessness, or changes in appetite or weight.

Her past medical history included hypertension, non-alcoholic fatty liver disease, and mixed anxiety and depressive disorder. She was a non-smoker and did not consume alcohol. There was no history of asbestos or industrial dust exposure. She did not report any family history of cancer. Her regular medications included ramipril, sertraline, and propranolol.

On examination, her vital signs were within normal limits. Mild tenderness was noted in the trapezius, thoracic, and lumbar spine and upper abdomen. The rest of the physical examination was unremarkable. Full blood count, liver and renal function tests, and C-reactive protein were all within normal limits, as shown in Table 1.

**Table 1:** Laboratory test results of the patient on admission and six months later Reference ranges are based on values provided by the hospital laboratory. Normal serum calcium and angiotensin-converting enzyme levels suggest the absence of active disease.

Test	On presentation	Six months later	Reference range
Sodium	142	142	133-146 mmol/l
Potassium	4.2	4.3	3.5-5.3 mmol/l
Urea	4.2	3.6	2.5-7.8 mmol/l
Creatinine	53	53	45-84 µmol/l
Total protein	77	74	60-80 g/l
Albumin	45	44	35-50 g/l
Bilirubin	13	9	≤21 µmol/l
Alkaline phosphatase	74	67	30-130 u/l
Alanine transaminase	38	32	10-49 u/l
White cell count	6.51	5.58	4.0-11.0 × 10⁹/l
Hemoglobin	133	128	120-150 g/l
Neutrophils	4.44	3.35	2.0-7.0 × 10⁹/l
Platelets	229	227	140-400 × 10⁹/l
Lymphocytes	1.06	1.4	1.0-3.0 × 10⁹/l
Eosinophils	0.34	0.33	0.02-0.50 × 10⁹/l
Angiotensin-converting enzyme	-	<8	20-95 u/l
Calcium	-	2.59	2.17-2.56 mmol/l
C-reactive protein	5	-	≤10 mg/l

**Figure 1:**
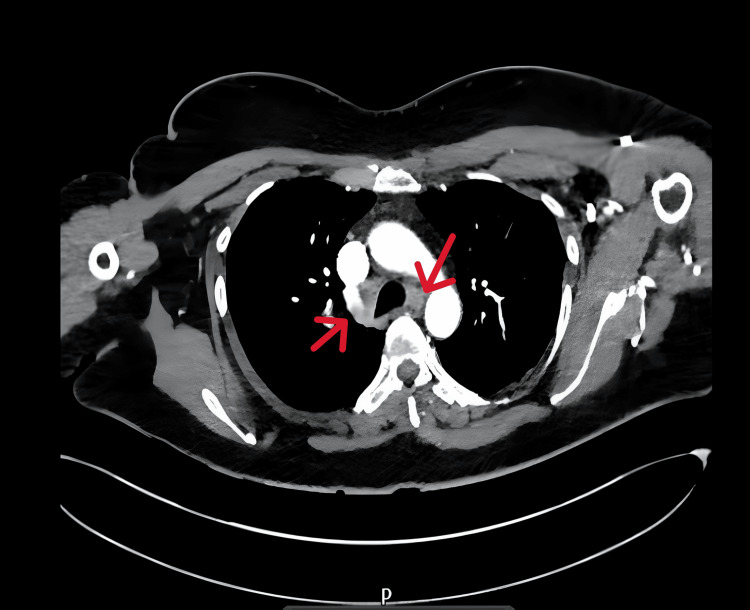
Sagittal CT of the chest showing bilateral mediastinal lymphadenopathy A key differential diagnosis for this radiological finding is sarcoidosis.

A CT scan from chest to pelvis was performed as part of the trauma protocol. It revealed enlarged mediastinal lymph nodes, indicated by red arrows in Figure 1, along with soft tissue densities in the left breast. 

The patient was discharged from the ED with a two-week wait referral to the breast clinic. Following a mammogram and ultrasound, stereotactic core biopsies were taken from the breast lesions, which revealed fibroadenoma. After discussion at the breast multidisciplinary team (MDT) meeting, the patient was discharged from breast care and referred to the lung MDT.

**Figure 2:**
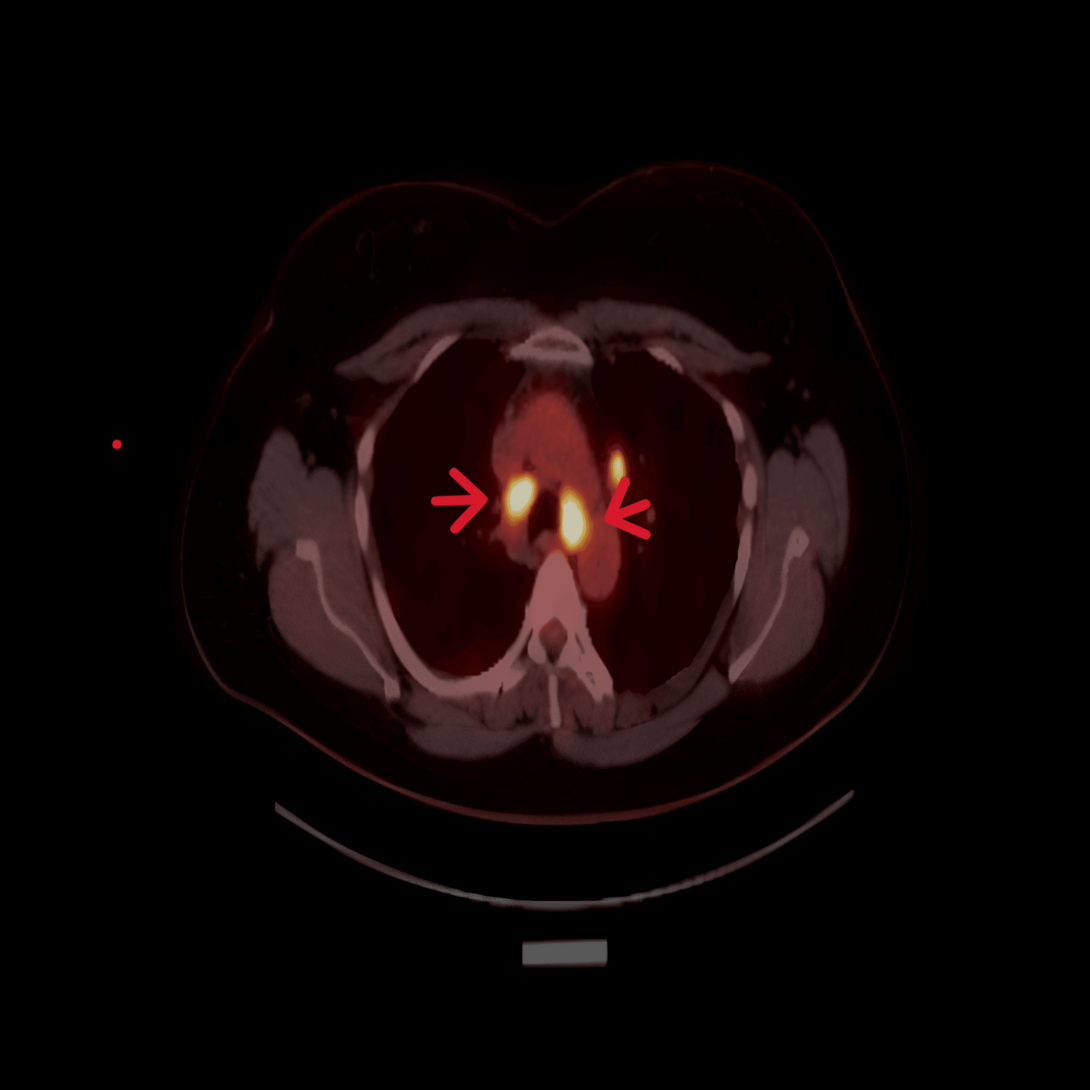
FDG PET scan showing avid fluorodeoxyglucose uptake in thoracic lymph nodes This finding, combined with avid uptake in a colonic lesion, raised suspicion of malignancy, which was later ruled out based on histopathology results.

**Figure 3:**
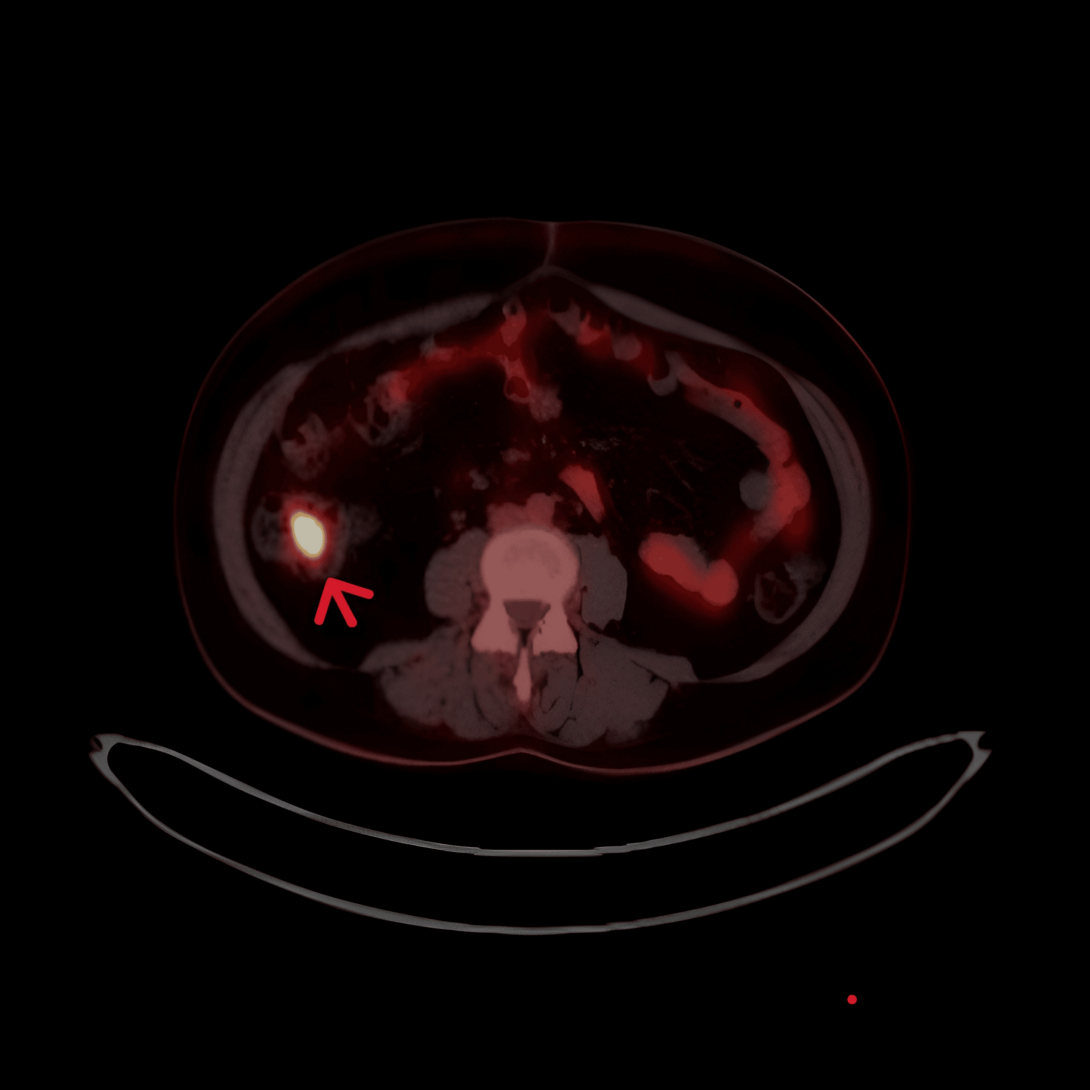
FDG PET scan showing avid fluorodeoxyglucose uptake in the ileocolic lymph node

As part of further investigations, a whole-body PET-CT scan was performed. It demonstrated avid uptake in the right cervical and bilateral thoracic lymph nodes (Figure 2), as well as focal high uptake in the ascending colon, raising suspicion for a colonic lesion. An associated 7 mm ileocolic lymph node was also identified (Figure 3).

**Figure 4:**
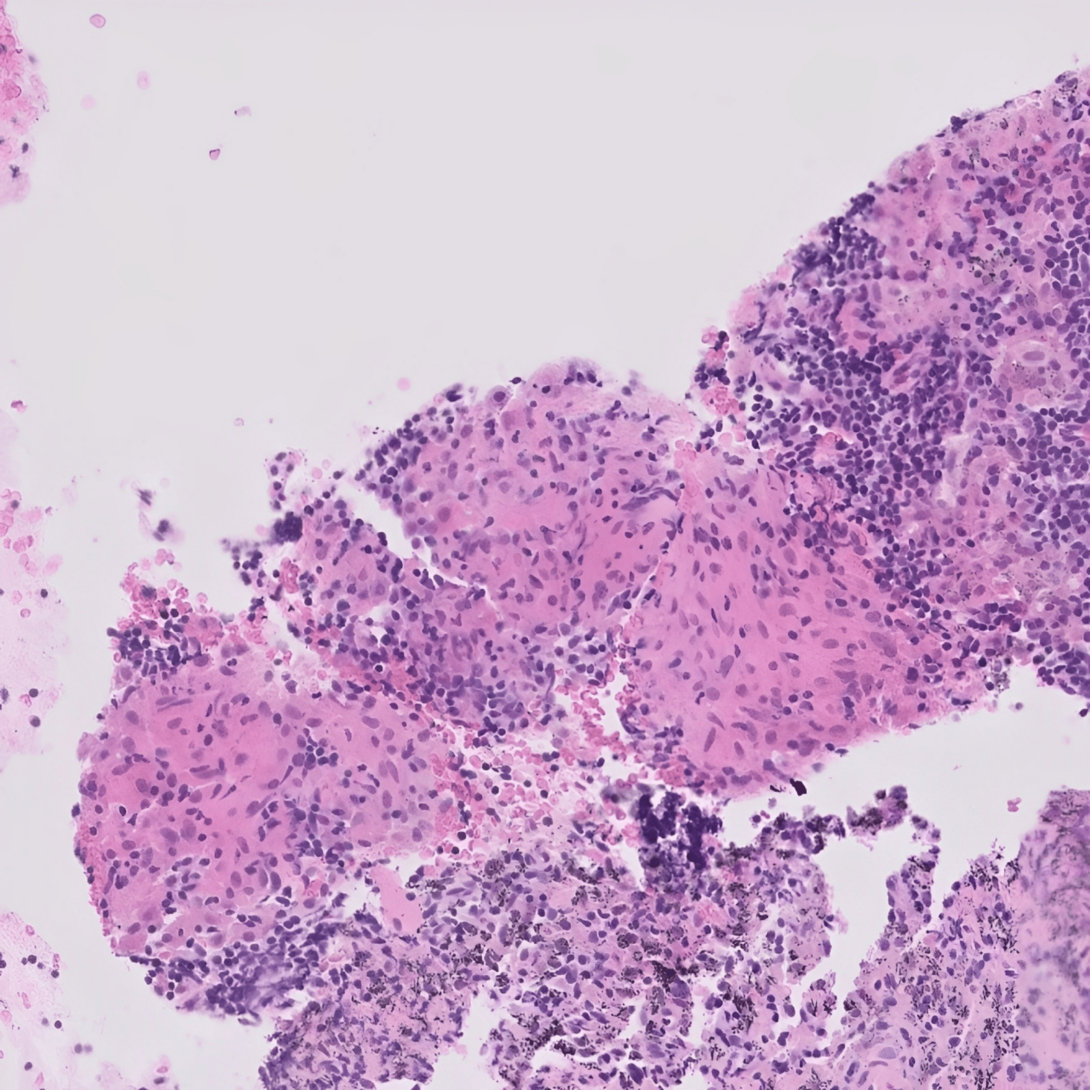
Endobronchial ultrasound-guided sample of a station 7 lymph node Histology shows lymphoid tissue with non-necrotizing granulomas, a hallmark feature of sarcoidosis.

A subsequent endobronchial ultrasound-guided biopsy of the subcarinal lymph nodes revealed lymphoid tissue with noncaseating granulomas, a hallmark feature of sarcoidosis (Figure 4).

**Figure 5:**
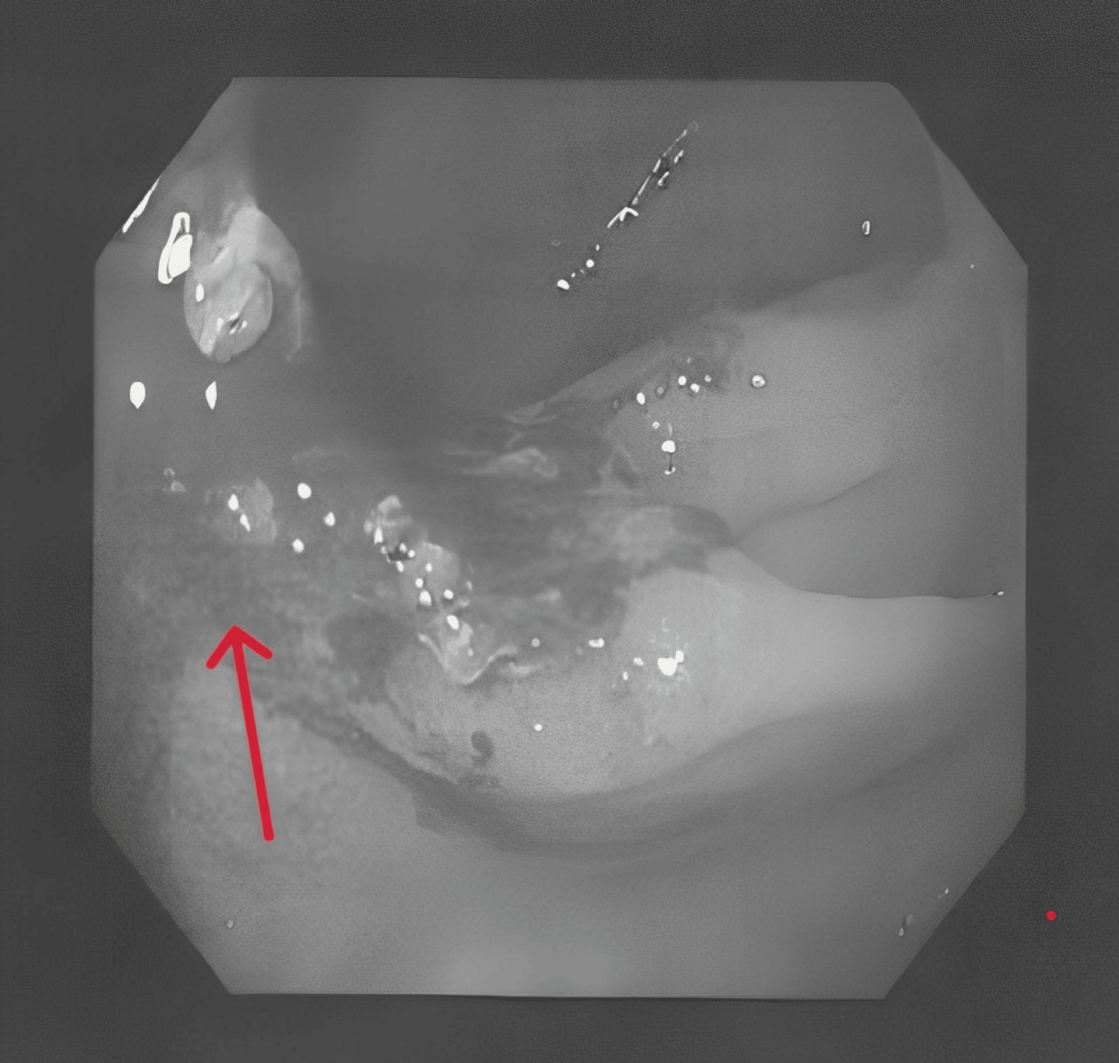
Colonoscopy image showing a colonic lesion Biopsy samples from the lesion revealed noncaseating granulomas, further supporting the diagnosis of sarcoidosis.

The colorectal team was involved, and a diagnostic colonoscopy was performed, which revealed a dysplastic lesion with an exophytic edge (Figure 5).

**Figure 6:**
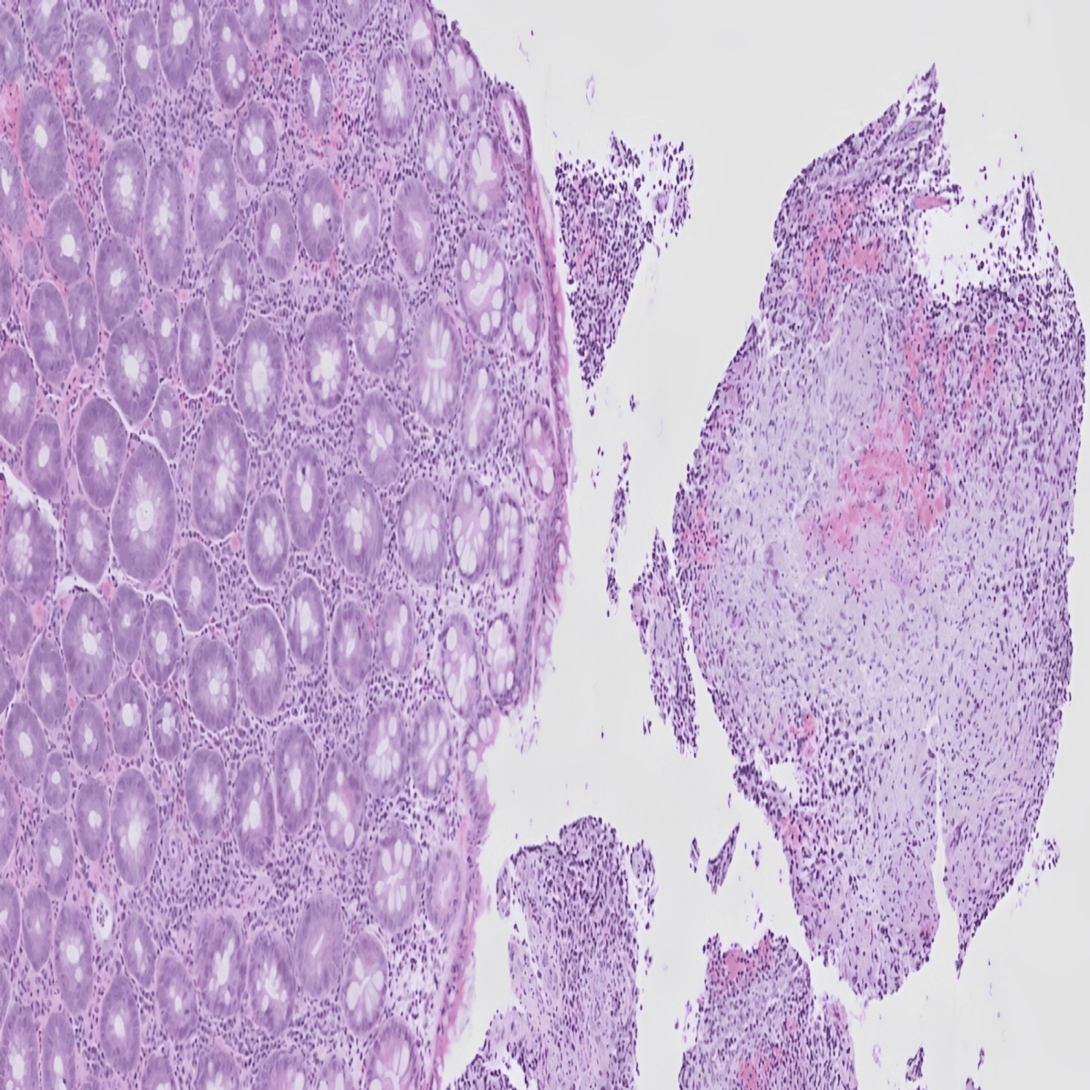
Large bowel mucosa showing ulceration and granulomatous inflammation consistent with sarcoidosis

Biopsy samples revealed ulceration with granulomatous inflammation (Figure 6). No acid-fast bacilli were identified in the Ziehl-Neelsen (ZN) stained section. Fungal organisms were not detected on diastase periodic acid-Schiff (DPAS) staining of the mediastinal lymph node biopsy. Serum calcium and ACE levels, both indicators of active disease, were within normal limits (Table 1).

## Discussion

Sarcoidosis is a rare, multisystem inflammatory disorder characterized by chronic granulomatous inflammation, with noncaseating granulomas observed on histopathological examination. The extent of organ involvement varies widely; pulmonary and thoracic lymph nodes are most commonly affected, whereas rarer manifestations include ocular, gastric, intestinal, hepatic, appendiceal, splenic, genitourinary, and neurosarcoidosis [1].

According to UpToDate, approximately 8% of patients present with extrapulmonary symptoms alone, without pulmonary involvement [1]. A few clinical manifestations are highly specific for sarcoidosis, such as Löfgren’s syndrome, lupus pernio, and Heerfordt’s syndrome (anterior uveitis, bilateral parotid gland enlargement, facial nerve palsy, and fever).

Establishing a definitive diagnosis of GI sarcoidosis can be challenging, as granulomas in the tubular structures of the GI tract and liver have several potential causes. Diagnosis relies on three key components: (1) identification of non-necrotizing granulomas in the affected organ; (2) exclusion of other causes of granulomatous inflammation, such as mycobacterial, fungal, and parasitic infections, inflammatory bowel disease, and Whipple’s disease; and (3) clinical, radiographic, and histopathological evidence of sarcoidosis in at least one other organ system [2].

Colonoscopy findings in GI sarcoidosis can vary, with reported appearances including nodules, ulcers, erythema, polyps, gastritis, erosions, small punctate bleeding, strictures, and aphthous ulcers. In patients from tuberculosis-endemic areas, a negative tuberculin skin test may support a diagnosis of sarcoidosis [2].

A literature search was conducted using BMJ Best Practice, Embase, and Ovid Medline, with search terms including “colon sarcoidosis”, “large intestine sarcoidosis”, “colon sarcoidosis involvement”, “large intestine sarcoidosis treatment”, “gastrointestinal sarcoidosis”, and “gastrointestinal tract”. Articles published between January 2005 and May 2025 were included. The search revealed that colonic involvement in sarcoidosis is rare, with only 30 cases reported in the literature [2]. GI sarcoidosis itself is extremely uncommon, occurring in only 0.1-0.9% of cases, with the stomach being the most frequently affected GI organ [3].

The most common presenting symptom in GI sarcoidosis is epigastric pain. Other reported symptoms include nausea, vomiting, diarrhea, and GI bleeding [3]. In a few cases, patients have presented with symptoms of intestinal obstruction [4-9]. Coexisting pulmonary disease was present in most reported cases of colonic sarcoidosis.

Sarcoidosis most commonly affects African American and Black populations; however, our case involved a patient of South Asian origin. Reported age ranges in the literature vary from the mid-twenties to the late seventies. One frequently noted association is gastroesophageal reflux disease (GERD), although it remains unclear whether GERD is a consequence of GI sarcoidosis or if both conditions coexist independently.

Our patient was found to have mediastinal lymphadenopathy, a colonic lesion with avid FDG uptake, and breast lumps, initially raising suspicion of metastatic malignancy. However, the absence of symptoms and normal physical examination findings did not support this clinically. One important differential diagnosis for noncaseating granulomas is Crohn’s disease, though this was deemed unlikely in our case, given the lack of supporting history and colonoscopic findings.

ZN staining of mediastinal lymph node and colonic biopsy samples did not reveal acid-fast bacilli, ruling out mycobacterial infection. Fungal organisms were also not identified on DPAS staining of mediastinal lymph node tissue, making fungal infection an unlikely cause. The patient was not tested for parasitic infections or Whipple’s disease, as there were no clinical, physical, or laboratory indications to suggest these conditions.

During the colorectal MDT discussion, while awaiting biopsy results from the colonic lesion and lymph nodes, a hemicolectomy was initially considered. However, based on the overall clinical picture and confirmed histopathology, this option was ultimately ruled out.

In active sarcoidosis, serum calcium and angiotensin-converting enzyme levels are typically elevated. In our patient, both values were within normal limits, suggesting the absence of active disease (Table 1). As she remained asymptomatic, no treatment was initiated, in line with current recommendations. For symptomatic patients, corticosteroids remain the first-line treatment. In cases where steroids are not tolerated or are ineffective, methotrexate, azathioprine, or infliximab may be considered [9,10]. Surgical intervention is generally reserved for obstructive complications.

Currently, there are no established guidelines for radiological follow-up in asymptomatic patients with incidental findings of sarcoidosis. Our patient was discharged with advice to return if she developed symptoms such as fever, weight loss, joint pain, altered bowel habits, rash, breathlessness, cough, or visual disturbances.

## Conclusions

Intestinal sarcoidosis remains a rare clinical entity. This case underscores the importance of considering alternative diagnoses in asymptomatic patients with radiological findings suggestive of malignancy. MDT involvement is essential for appropriate evaluation and management. A definitive diagnosis requires a combination of imaging, histopathological confirmation, and exclusion of other causes of noncaseating granulomas. Further clinical studies are needed to explore a potential association between GI sarcoidosis and GERD. Additionally, clear guidelines are needed for the follow-up and monitoring of patients, particularly those with extrapulmonary manifestations.
